# The impact of SLMTA in improving laboratory quality systems in the Caribbean Region

**DOI:** 10.4102/ajlm.v3i2.199

**Published:** 2014-11-03

**Authors:** Giselle Guevara, Floris Gordon, Yvette Irving, Ismae Whyms, Keith Parris, Songee Beckles, Talkmore Maruta, Nqobile Ndlovu, Rachel Albalak, George Alemnji

**Affiliations:** 1 US Centers for Disease Control and Prevention, Caribbean Regional Office, Barbados; 2 African Field Epidemiology Network, Caribbean Office; 3 Princess Margaret Hospital, Bahamas; 4 Ladymeade Reference Unit, Barbados; 5 African Field Epidemiology Network, Uganda; 6 Clinton Health Access Initiative, Botswana

## Abstract

**Background:**

Past efforts to improve laboratory quality systems and to achieve accreditation for better patient care in the Caribbean Region have been slow.

**Objective:**

To describe the impact of the Strengthening of Laboratory Management Toward Accreditation (SLMTA) training programme and mentorship amongst five clinical laboratories in the Caribbean after 18 months.

**Method:**

Five national reference laboratories from four countries participated in the SLMTA programme that incorporated classroom teaching and implementation of improvement projects. Mentors were assigned to the laboratories to guide trainees on their improvement projects and to assist in the development of Quality Management Systems (QMS). Audits were conducted at baseline, six months, exit (at 12 months) and post-SLMTA (at 18 months) using the Stepwise Laboratory Quality Improvement Process Towards Accreditation (SLIPTA) checklist to measure changes in implementation of the QMS during the period. At the end of each audit, a comprehensive implementation plan was developed in order to address gaps.

**Results:**

Baseline audit scores ranged from 19% to 52%, corresponding to 0 stars on the SLIPTA five-star scale. After 18 months, one laboratory reached four stars, two reached three stars and two reached two stars. There was a corresponding decrease in nonconformities and development of over 100 management and technical standard operating procedures in each of the five laboratories.

**Conclusion:**

The tremendous improvement in these five Caribbean laboratories shows that SLMTA coupled with mentorship is an effective, user-friendly, flexible and customisable approach to the implementation of laboratory QMS. It is recommended that other laboratories in the region consider using the SLMTA training programme as they engage in quality systems improvement and preparation for accreditation.

## Introduction

Improving laboratory quality systems and attaining accreditation are important benchmarks in National Health Laboratory practice, as accreditation is a process that gives formal recognition of the technical competence of a laboratory to perform specific tests.^1^ In many cases, the added value of accreditation far outweighs the necessary investment in human resources, finances and time, since it is an independent method of determining and monitoring laboratory performance, whilst assuring the validity of the results to the users.^2,3^

Implementation of laboratory Quality Management Systems (QMS) and achievement of accreditation amongst laboratories in the Caribbean Region has been limited. Available data report only three accredited government-owned or public clinical laboratories in the Caribbean as of 2011.^4^ Over the years, many Caribbean laboratory staff have been provided with information on QMS and accreditation in various forms, including training, conferences, meetings and printed material. However, using this knowledge collectively and developing a comprehensive plan in order to address quality gaps and begin the journey toward accreditation have been challenging. During a preliminary laboratory needs assessment survey conducted in 2009, laboratory managers and other stakeholders discussed the problems of an undertrained laboratory workforce, the lack of motivation and, most importantly, the perception that the quality improvement process was cumbersome.^4^ The need to put strategies in place to eliminate these hindrances as soon as possible was emphasised. The recommendation was that a more user-friendly, stepwise approach to quality systems implementation, in combination with task-based training tools to improve staff knowledge, could lead to more substantial improvement in quality systems.

The Strengthening Laboratory Management Toward Accreditation (SLMTA) programme was launched in 2009 and has been implemented in 47 countries worldwide.^5^ It is a management training programme that utilises a series of workshops interspersed with on-site projects designed to improve laboratory quality. Evidence from other settings has shown that the SLMTA training programme yields observable and measurable laboratory improvements.^6^ Furthermore, the training empowers laboratory staff and enhances management’s ability to improve their own laboratories by making use of existing resources.^7^ A laboratory quality improvement mentorship intervention programme in Lesotho that incorporated the SLMTA training and a stepwise approach to accreditation preparedness has resulted in significant measurable improvements in the quality of enrolled laboratories over a period of 12 months.^8^

The reauthorisation of the US President’s Emergency Plan for AIDS Relief (PEPFAR II) in 2008 resulted in the establishment of the PEPFAR Caribbean Regional Program and the development of the PEPFAR Partnership Framework with 12 Caribbean countries (Barbados; Trinidad and Tobago; Belize; Suriname; Jamaica; the Bahamas; St. Lucia; St. Vincent and the Grenadines; Grenada; Antigua and Barbuda; St. Kitts and Nevis; and Dominica). Since then, the PEPFAR laboratory-strengthening working group has worked closely with the Ministries of Health (MOHs) in these countries to improve the quality and reliability of laboratory results and to offer basic testing services for persons living with HIV. The need to engage laboratories in these countries in quality improvement and accreditation was identified very early during this collaboration when it became apparent that laboratory services, systems and infrastructure in the region were weak, with various populations lacking access to timely, low-cost and high-quality laboratory services.^4^

With the aim of improving laboratory quality in the region, the US Centers for Disease Control and Prevention (CDC) Caribbean Regional Office Laboratory Team, the International Laboratory Branch of the Division of Global HIV/AIDS at CDC Atlanta and the African Field Epidemiology Network (the laboratory implementing partner) collaborated to research options for effective laboratory quality improvement. The decision was made to use the SLMTA training programme, coupled with the World Health Organization Regional Office for Africa’s (WHO AFRO) Stepwise Laboratory Quality Improvement Process Towards Accreditation (SLIPTA) checklist, along with mentorship, in order to improve the quality systems of five laboratories in four of the Caribbean Partnership Framework countries. This article discusses improvements in the laboratory quality systems during the 18-month implementation of the SLMTA training programme and mentorship in these laboratories.

## Research method and design

### Advocacy strategy with governments

At the initiation of the regional laboratory strengthening activities, following the signing of the PEPFAR Caribbean Regional Partnership Framework in 2010, key sensitisation meetings were held with policymakers and other stakeholders in each of the four countries to highlight the need, importance and advantages of improved laboratory quality systems and accreditation. These meetings included Chief Medical Officers, Permanent Secretaries, laboratory directors and other regional partners. In addition to discussing an overall strategy for collaboration and strengthening of the entire laboratory health system, a presentation was made highlighting the stepwise approach toward accreditation, the SLMTA training programme and the use of mentors as innovative approaches to implementing quality systems and eventually achieving accreditation.

The proposed strategy for laboratory strengthening began by engaging the national reference laboratories in each of the four selected countries. Although each laboratory was unique in its operation, size and workload, it was agreed that the challenges faced were similar and they would, therefore, all benefit from the proposed interventions. To ensure buy-in and to highlight the need for providing additional resources to address the deficiencies previously identified during the laboratory needs assessment survey in 2009 and the subsequent baseline audits in 2011, key senior officials from the human resources, procurement and maintenance departments of the MOHs and hospitals were invited to attend the audit debrief meetings in their respective countries.

### Laboratory audits

Periodic audits spanning three to four days were conducted in each of the five national reference laboratories by experienced auditors using the SLIPTA checklist. The SLIPTA programme uses a stepwise accreditation preparedness scheme that recognises laboratories according to their level of compliance with the the international standard ISO 15189 – Medical Laboratories – Particular requirements for quality and competence. The results of the laboratory audits were reported for each of the 12 sections of the checklist covering the 12 quality systems essentials (CLSI GP 26-A3 [2004]), including 111 main items for a total of 258 possible points (Table 1). The score obtained by each laboratory indicates the level of performance, which determines the star rating from 0 to five stars.

**TABLE 1:** SLIPTA scoring system for laboratories.

Accreditation Checklist	Total Points
Section 1: Documents and Records	25
Section 2: Management Reviews	17
Section 3: Organisation and Personnel	20
Section 4: Client Management and Customer Service	8
Section 5: Equipment	30
Section 6: Internal Audit	10
Section 7: Purchasing and Inventory	30
Section 8: Process Control and Internal and External Quality Assessment	33
Section 9: Information Management	18
Section 10: Corrective Action	12
Section 11: Occurrence/Incident, Management and Process Improvement	12
Section 12: Facilities and Safety	43
**Total score**	**258**

SLIPTA, Stepwise Laboratory Quality Improvement Process Towards Accreditation; WHO AFRO, World Health Organization Regional Office for Africa.

0 Stars, (0 – 141 pts) < 55%; 1 Star, (142 – 166 pts) 55% – 64%; 2 Stars, (167 – 192 pts) 65% – 74%; 3 Stars, (193 – 218 pts) 75% – 84%; 4 Stars, (219 – 243 pts) 85% – 94%; 5 Stars, (244 – 258 pts) ≥ 95%.

**FIGURE 1:**
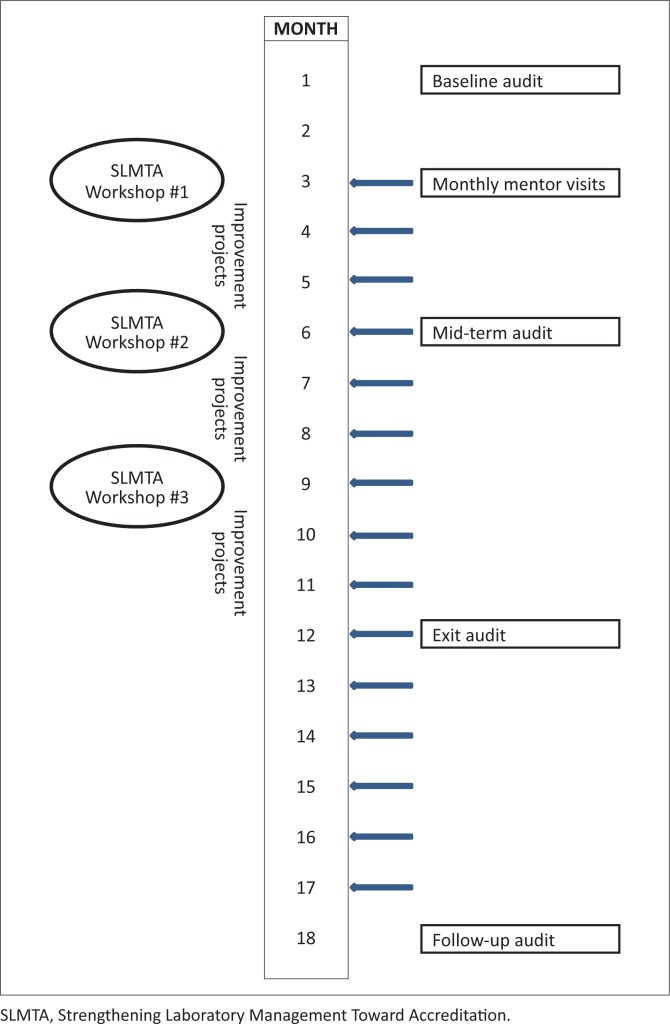
Laboratory strengthening implementation model for the Caribbean Region.

The audits were conducted in each of the five participating laboratories at baseline, after six months (mid-term audit), after 12 months (exit audit) and after 18 months (follow-up audit) to ensure continuous monitoring of the laboratories and their performance (Figure 1).

Each laboratory audit began with an introductory meeting convening the laboratory director and departmental heads in order to summarise the proposed audit plan which would be used to identify areas for improvement. At the end of the audit, a formal debrief meeting was held with laboratory management, technical staff and key persons from the MOH and hospital whose responsibilities affect the smooth functioning of the laboratories. After the baseline audit, a customised quality system implementation plan was developed in order to outline the nonconformities found, recommendations for follow-up actions, responsible persons, timeline for completion and status (Table 2).

**TABLE 2:** Example of quality systems implementation plan.

Nonconformity	Recommendations/Comments	SLIPTA Checklist Questions	ISO 15189 Reference	Timeline	Responsible Person	Status
Quality manual is in draft form.	Need to review and authorise quality manual.	1.1	4.2.4 and 4.2.3	6 months	Quality Manager	Pending
There are no quality objectives for the Quality Management System, nor a statement of management commitment.	The laboratory needs to clearly define its quality objectives as required by ISO 15189 and demonstrate proof of management commitment.	1.1	4.2.3 part(c)	2 months	Laboratory Director	Pending
There was no evidence of follow up and corrective action for unsatisfactory EQA results.	The lab needs to implement root cause analysis and corrective actions for EQA results.	8.12	4.2.2, 5.6.4, 5.6.5, 5.6.7	2 months	Quality Manager	Pending
Validation/verification records were not available for some equipment, e.g., Haematology and Chemistry.	The laboratory to procure validation panels and conduct the validation exercise. Or request that manufacturers provide validation reports.	5.2	5.5.2	3 months	Quality Manager, Department Heads	Partial

SLIPTA, Stepwise Laboratory Quality Improvement Process Towards Accreditation; ISO, International Organization for Standardization; EQA, external quality assessment.

Throughout the programme the laboratories were audited at approximately six-month intervals, which allowed them to monitor their continued progress and update the quality system improvement plans originally developed at the baseline audit. The list of nonconformities found at the previous audit was also comprehensively reviewed and updated to determine the number of completed corrective actions over the period. Open nonconformities were assigned for further follow-up by the laboratory and its management.

Exit audits were conducted using the SLIPTA checklist three months after the last SLMTA workshop concluded (12 months after baseline). A follow-up audit was then conducted six months later to evaluate the longer-term effectiveness and sustainability of the programme. These audits allowed laboratories to determine their level of progress from the baseline to exit of the SLMTA training and mentorship programme.

#### SLMTA workshops

The SLMTA training programme was implemented as a series of three workshops which began in May 2011 and were conducted approximately three months apart (Figure 1). A total of 24 participants (three to five per laboratory) from across the five focus laboratories were chosen based on the size of their laboratory and the testing needs of each country. These included staff from the various departments, (i.e., Chemistry, Blood Bank, Serology, etc.), as well as the quality manager or designee. Participants were required to develop improvement projects and complete them during the hiatus between workshops. The improvement projects were generally chosen based on the areas of nonconformity indicated in the laboratory’s individualised quality systems implementation plan along with the needs of the laboratory at the time. Each participant presented a summary of their completed improvement projects at the subsequent workshop, including the baseline data collected, the measure of progress within the study period and the challenges experienced during project execution. Final improvement projects were presented orally by each participant and graduation certificates were awarded to them in the presence of officials from the MOH and the hospitals in order to highlight the importance of this event to the process of accreditation preparedness.

#### Mentorship for the laboratories

Each of the engaged laboratories was assigned a mentor to assist in developing and establishing their QMS by providing technical assistance and coaching on implementing the improvement projects from the SLMTA training. Three fulltime mentors were used for this activity across the five laboratories. Each mentor had at least 10 years of experience in laboratory technology and development of QMS.

During the first few months of the programme, the mentors spent approximately one week each month embedded in the assigned laboratory. After approximately six months, the length of each mentor’s assignment was increased to two or three weeks, depending on the needs of the laboratories at that time.

Six-week mentorship action plans were developed to give direction to both the laboratory and the mentor, allowing for measurement of progress over the specific six-week period (Table 3). Since the mentor was physically on-site for only part of the six-week period, the laboratory had a period of self-management during which time they communicated with the mentor via email, internet conferencing and telephone. All management and technical procedures produced during the assignments were forwarded to the laboratory directors or department directors for final approval.

**TABLE 3:** Example of six-week mentorship action plan for a laboratory.

Week	Routine Activities	SLMTA Follow-up Activity
1	Using the ISO 15189, meet with Quality Manager to ensure policies and procedures are revised or created; review findings with supervisors.	Facilitate SLMTA activities to support equipment management.
2	Develop schedule for internal reviews with the Quality Manager or department directors. Conduct training on root cause analysis. Review the document archival system and revise accordingly.	Facilitate SLMTA activities to support procurement and inventory.
3	Introduce quality objectives, indicators and improvement projects. Develop a schedule for internal audits, and conduct training on internal auditing with Quality Manager.	Facilitate SLMTA activities to support process control.
4	Develop checklist(s) to guide the review and authorisation of documents. Review data on quality indicators and work with department heads to develop quality improvement activities.	Review checklist items 5.0; 7.0; 8.0 and 12.0 in all departments.
5	Review the Safety Manual against the requirements of ISO 15190. Revise schedule for staff meetings; perform desktop review of procedures developed/revised in week 1.	Facilitate SLMTA activities to support safety.
6	Discuss with Quality Manager annual management reviews (planning and follow up).	Conduct audits against ISO 15189 for process control, equipment, safety, procurement and inventory.

Note: Activities cutting across the six weeks: Conduct training on revised procedures from week one. With the Quality Manager and section heads develop action plans following all internal reviews and practise using root cause analysis techniques and completion of corrective and preventive action forms.

SLMTA, Strengthening Laboratory Management Toward Accreditation; ISO, International Organization for Standardization.

## Results

**FIGURE 2:**
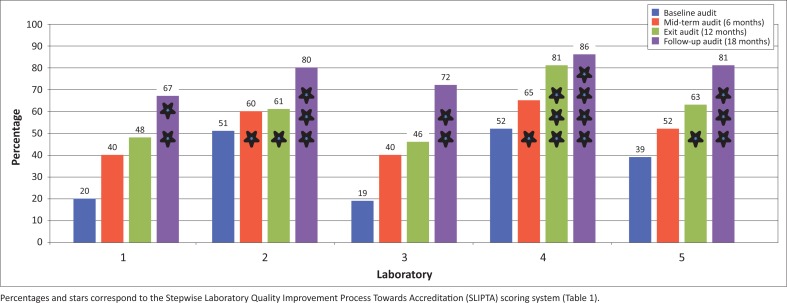
Improvement in implementation of the laboratory quality systems and stars attained over 18 months.

At the baseline audits the laboratory scores ranged from 19% to 52%, corresponding to 0 stars (Figure 2). Scores increased steadily throughout the programme and by 18 months each laboratory had improved, with three of the laboratories more than doubling their baseline scores. One laboratory reached four stars on the five-star scale, two attained three stars and the remaining two laboratories each attained two stars. Of this group, one laboratory achieved accreditation through the College of American Pathologists (CAP) in September 2013; meanwhile three others have applied for accreditation and are preparing for the assessment within the next few months.

**FIGURE 3:**
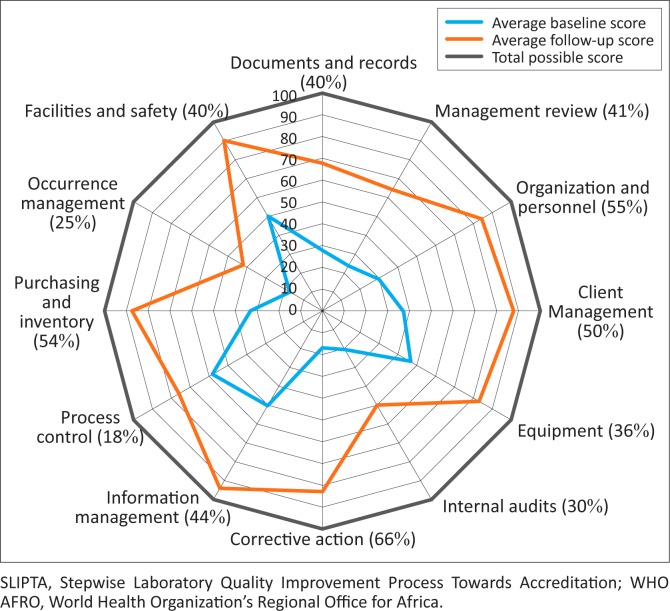
Average performance improvement of all laboratories across the 12 sections of the WHO AFRO SLIPTA checklist.

Figure 3 shows the average percentage improvement across the five laboratories for each of the 12 sections of the checklist (i.e., the 12 quality system essentials), measured as the difference between the baseline and follow-up score after 18 months. The greatest improvements were in corrective action (66%), organisation and personnel (55%) and purchasing and inventory (54%). The sections showing the least improvement were process control (18%), occurrence management (25%), internal audits (30%) and equipment (36%). Average final absolute scores were > 60% for all areas except occurrence management and internal audits.

Overall, between 141 and 735 Standard Operating Procedures (SOPs) were completed and approved for each laboratory over the 18-month period (Table 4), leading to an average increase on the checklist of 40% from the baseline score in the area of documents and records (Figure 3).

**TABLE 4:** Number of standard operating procedures (SOPs) completed for the 5 laboratories.

Laboratory	Size of Laboratory*	Management SOPs	Technical SOPs	Total SOPs Produced
1	Large	29	176	205
2	Large	60	396	456
3	Medium	169	123	292
4	Large	303	432	735
5	Small	53	88	141

* Laboratories were categorised according to number of staff as follows: Small, < 20; Medium, 20–30; Large, ≥ 31.

**FIGURE 4:**
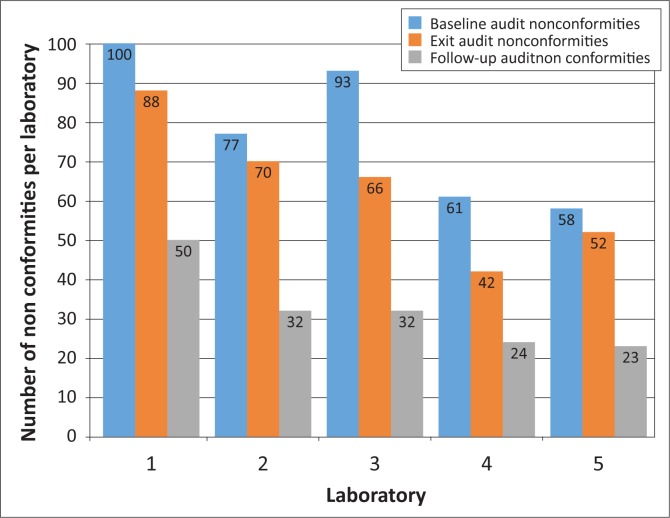
Change in number of nonconformities per laboratory over time.

Improvement in each laboratory can also be measured by the change in the number of identified nonconformities (Figure 4). Nonconformities decreased by more than half during the intervention period. For each laboratory this translated into at least a 50% decrease in outstanding nonconformities over the entire implementation period.

### Case studies

Each participant enrolled in the SLMTA training programme was required to choose, plan and execute at least three improvement projects over the duration of the programme. SLMTA trainers provided tools, techniques and examples in order to guide participants to design effective projects within their laboratory, whilst mentors provided implementation support. As a result of these projects, tangible improvements were observed in the QMS and overall operations of the laboratories. Two high-impact projects are presented here as case studies:

#### Case Study 1 – Inventory management

Laboratory 4 has three store rooms containing hundreds of supplies from various vendors. The baseline audit showed that management of stock was a challenge within this facility, with frequent stock-outs, lack of proper tracking forms in the storage areas and increased borrowing from other laboratories. Upon investigation, factors such as unpredictable patient-testing workload, delivery delays and back-order issues consistently affected the supply levels. These issues were exacerbated by the poor record keeping and lack of an organised inventory management system, preventing effective forecasting.

A key recommendation to the SLMTA trainee was to put a system in place to ensure sufficient stock levels of all supplies. Hence, an improvement project was designed to enhance inventory management in all areas of the system, with the overall objective to reduce stock-outs to less than 5% within a four-month period. To achieve this objective, all staff were briefed on the project, including their specific roles in the success of the intervention. The following data collection and monitoring tools were developed: Expired Reagent Record Log; CARDEX for Storage Areas; Order Form; Laboratory Inventory Card; Inventory Control Colour Chart; Laboratory Loan Form; Requisition Order Code Form; Quotation Request Form; Receiving and Inspection Investigative Checklist; Regular Receiving and Inspection Log; Refrigerator CARDEX; Section Grading Card; Inventory and Usage Pattern Data Collection Log; and a Laboratory Stores Task Assignment Checklist.

**FIGURE 5:**
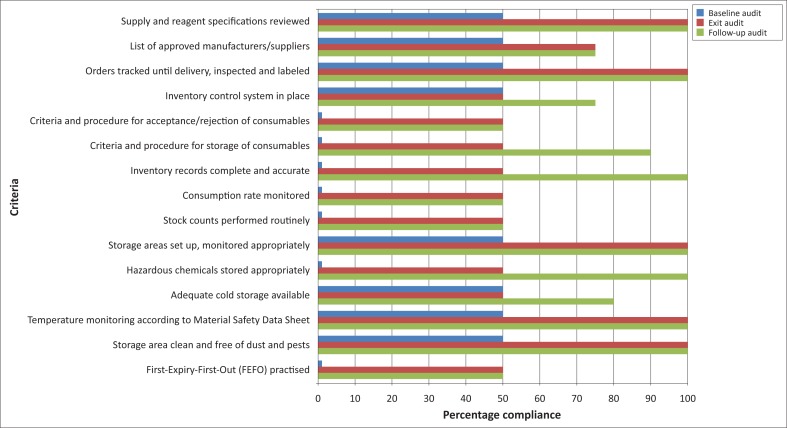
Improvement in laboratory stock management in Laboratory 4.

During the improvement project, 15 quality indicators were monitored (Figure 5). The results showed that seven of the 15 areas either maintained or achieved 100% compliance, whilst two other areas achieved 90% and 80% compliance over the baseline results. Other areas achieved appreciable improvements (Figure 5). Overall stock-outs were reduced to 5% as a result of the general improvements in the system.

#### Case Study 2 – Improving documents and records management in the microbiology laboratory

Laboratory 2 has had problems managing quality system documents and associated manuals in their microbiology section. This has resulted in limited progress toward achieving accreditation and difficulty in training new staff in the department.

An improvement project was designed to address document and records management. A team of key organisational individuals was convened to work together on the development of the QMS. This critical step helped to gain support for the project throughout the various sections in the department. Section leaders had the ultimate responsibility of designating and distributing the assignments within their sections. The documents and records were grouped into four categories: Technical SOPs; Management SOPs; Logs and Checklists; and Equipment (including the Equipment list, Preventative Maintenance logs and SOPs for each item of equipment).

**FIGURE 6:**
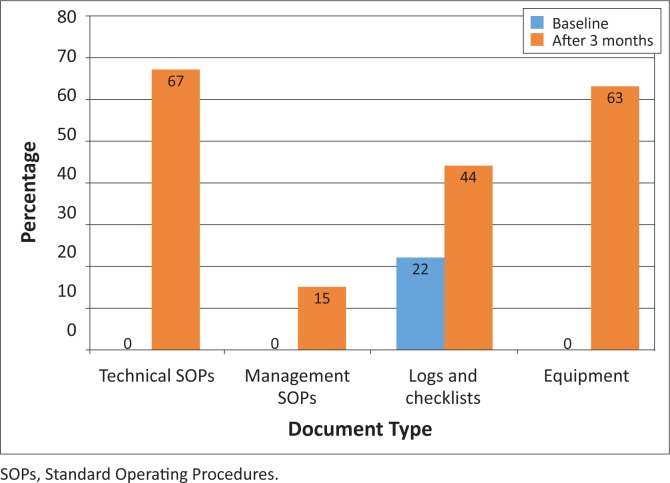
Improvement in documentation for Laboratory 2.

Figure 6 depicts the level of improvement in documentation after three months of this intervention. Technical SOPs showed the highest level of improvement, from 0% to 67%, closely followed by Equipment documentation, from 0% to 63%; the least improvement was in the Management SOPs.

## Discussion

Although diverse in its geography, people, size and economy, the Caribbean Region shares a common challenge in achieving accreditation of its medical laboratories. Previous didactic training programmes introduced laboratory staff to the basic quality management principles and the existence of the ISO 15189 standard. Despite this knowledge, limited progress was seen. An approach that encompassed SLMTA training, a stepwise evaluation process and mentorship has resulted in tremendous improvement in the quality systems of five national laboratories in four countries of the region within an 18-month period, one of them having attained accreditation. Several factors may have contributed to the successes:

### Early engagement of key stakeholders

Key to the success of global health interventions is full engagement of decision makers in the process from the beginning. In particular, facilitating meetings of policy makers – Permanent Secretaries, Chief Medical Officers and top management officials of the hospital – along with technical staff, in order to identify challenges and opportunities to resolve nonconformities was important for this project, since these individuals subsequently provided the laboratories with the support and resources needed to ensure timely improvement of the quality systems. Endorsement by top management for laboratory systems strengthening activities has proven to be important for the success of this stepwise approach.

### An implementation roadmap

The process of accreditation can appear to be daunting, as extremely high levels of compliance with the quality requirements are essential for a successful assessment and a passing score. For a laboratory without an effective QMS, identifying challenges and developing a quality improvement plan can seem like an insurmountable goal, which can lead to demotivation and subsequent inaction. The use of a stepwise improvement process, along with specialised guidance documents, has been shown to provide laboratory stakeholders with a clearer path toward quality systems improvement and accreditation.^1^ Caribbean laboratory directors and managers emphasised that past laboratory assessments and training did not provide them with a structured roadmap to assist in implementation; as a result, the majority of these laboratories did not initiate the process of QMS development and implementation.^4^

The SLIPTA checklist was used to conduct an initial gap analysis in the participating laboratories, leading to the development of an implementation plan, which provided direction for improving the laboratory QMS. This plan outlined the process to be taken and the indicators that would be used to measure tangible progress and outcomes over time. Everyone involved, including hospital management, was assigned specific tasks relating to their functions and roles, with key deliverables and solid deadlines. Use of the stepwise evaluation method enabled recognition of incremental improvements at each audit throughout the process, providing added motivation to all the staff. The scores achieved at each audit highlighted the status attained and the progress that the laboratories had made in building an effective QMS, in eliminating nonconformities and in their readiness for accreditation.

### Structured improvement approach

Prior approaches to laboratory strengthening in the region focused mainly on mass sensitisation to and training on the ISO standards and quality management basics, but not on implementation. In some cases the persons trained had not previously been exposed to the principles of continuous quality improvement, total quality management, or development of a quality system specifically for the laboratory. The SLMTA programme taught the enrolled laboratories how to change the way they approached quality management and their daily operations. The programme also provided user-friendly tools that allowed staff to work more efficiently, as evidenced by their improved star ratings after 18 months.

An important component of the SLMTA training is the improvement projects developed and implemented by the trainees. This promoted a culture of systematic problem solving and a strategic approach to the application of quality system requirements. These projects and their measureable results served as a tool for the laboratory to advocate with management and policymakers for continued support. With the changing economic priorities and limited resources in these developing countries, it was critical to document the impact of any quality improvement and accreditation preparations, so as to demonstrate for stakeholders that the benefits outweigh the costs.^2^ In the case of these Caribbean laboratories, nonconformities were drastically reduced, with corresponding improvement in each of the quality management systems. For example, a 66% improvement was observed in the laboratories’ ability to perform corrective actions. A similar SLMTA intervention in Lesotho^6^ reported a 34% improvement in corrective action application over an 11-month period.

### Mentorship

According to Maruta, Rotz and Peter, ‘a laboratory mentoring program can be an important way to establish and solidify quality management systems and to help laboratories achieve accreditation goals’.^9^ The presence of the mentors in this programme served two main purposes. Firstly, mentors provided needed technical assistance in order to aid the laboratory in the development and finalisation of the QMS documentation. It has been documented that a strong foundation for quality assurance begins with development of a quality manual, SOPs and test methods, since they serve as a guide for both implementing and enhancing the quality system.^10^ The mentors played a critical role in bridging the gap between what was learnt in the workshops and what was implemented within the laboratories, drawing the team together to develop a strategy and guiding them to address the existing issues. For example, the majority of laboratory staff initially reported that their quality documents were delayed in the process of development for six or more months. The reduction in nonconformities recorded in these laboratories can be directly linked to the increase in the number of documents developed, completed and implemented as a result of the technical assistance provided by the mentors.

Secondly, mentors alleviated fears associated with preparation for accreditation and acted as a catalyst for enhanced communication. With the mentors present, communication improved greatly amongst the laboratory staff, laboratory management and medical staff. Management was more open to presentations and discussions with the laboratory staff, since these consultations were centered on actual data, nonconformance reports and demonstrated improvement. Staff often planned management review meetings whilst the mentor was on site, allowing the mentor to help facilitate communication with upper management and showcase the improvement in the laboratory QMS as a result of the interventions.

### Key challenges and recommendations

The Caribbean Region is made up of small island nations with most country populations in the range of hundreds of thousands. Ensuring a sufficient number of well-qualified laboratory workers is an ongoing challenge, exacerbated by high levels of attrition as staff that have benefitted from government-supported training leave the public sector for more lucrative jobs in the private sector, either locally or overseas. Thus the remaining staff are overworked, reducing the amount of time available for training and quality improvement activities. There is also a shortage of qualified mentors who can provide the needed support to laboratories engaged in quality improvement efforts and accreditation preparation. These personnel challenges limit the laboratories’ opportunities for development of QMS and achievement of laboratory accreditation. Encouraging governments in the region to prioritise health system–strengthening strategies that lead to staff development and retention would benefit not only laboratories, but the health system overall.

One of the main logistical challenges faced in this programme stemmed from the use of mentors based in different countries, who were required to travel by air to provide on-site support. Thus, considerable funds needed to be invested and intervention was sometimes delayed because of travel issues. Establishment of a cadre of in-country or regional SLMTA trainers and mentors would build local capacity and help reduce programme costs, especially as the programme expands.

The momentum achieved through success of the SLMTA programme in these five laboratories must now be directed to further improvements in these laboratories, as well as expansion of the programme throughout the region. One of the participating laboratories recently achieved accreditation from CAP and three more have subsequently applied for accreditation, as a direct result of the training and technical assistance received in the SLMTA programme. The remaining laboratory will continue to be monitored by means of SLIPTA audits, whilst preparing actively for accreditation in the near future.

Introduction and implementation of the SLMTA programme in the Caribbean Region has been made possible by funding from the PEPFAR programme; however, there is now a need to internalise the programme and transition it to local governments and other donors in order to facilitate expansion and ensure sustainability.

### Conclusion

Quality management interventions in the Caribbean over the past 10 years had resulted in few improvements in the overall laboratory quality infrastructure, as evidenced by the low performance scores achieved at baseline audits and the limited number of previously-accredited laboratories in the region. A change of approach was thus needed in order to increase these numbers and put more laboratories on the path to accreditation. Implementation of the SLMTA and mentorship approach in several laboratories in the region has achieved tangible improvements in QMS development and overall quality within a very short period. Continued improvement in these laboratories and expansion of this programme to other laboratories in the region are recommended.

Sustained improvement will require government funds to be invested in training resources, including development and establishment of local mentorship programmes. Our results strongly support the growing body of evidence indicating that the SLMTA training programme is an important tool to empower laboratory staff, enhance management competence and achieve observable and measurable results for improved laboratory quality.

## References

[1] Gershy-Damet GM, Rotz P, Cross D, et al The World Health Organization African region laboratory accreditation process: Improving the quality of laboratory systems in the African region. Am J Clin Pathol. 2010;134(3):393–400. http://dx.doi.org/10.1309/AJCPTUUC2V1WJQBM 20716795 10.1309/AJCPTUUC2V1WJQBM

[2] Zeh CE, Inzaule SC, Magero VO, et al Field experience in implementing ISO 15189 in Kisumu, Kenya. Am J Clin Pathol. 2010;134(3);410–418. http://dx.doi.org/10.1309/AJCPZIRKDUS5LK2D 20716797 10.1309/AJCPZIRKDUS5LK2D

[3] Peter TF, Rotz PD, Blair DH, et al Impact of laboratory accreditation on patient care and the health system. Am J Clin Pathol. 2010;134(4):550–555. http://dx.doi.org/10.1309/AJCPH1SKQ1HNWGHF 20855635 10.1309/AJCPH1SKQ1HNWGHF

[4] Alemnji GA, Branch S, Best A, et al Strengthening national laboratory health systems in the Caribbean region. Glob Public Health. 2012;7(6):648–660. http://dx.doi.org/10.1080/17441692.2012.670861 22519703 10.1080/17441692.2012.670861

[5] Yao K, Maruta T, Luman ET, Nkengasong JN The SLMTA programme: Transforming the laboratory landscape in developing countries. Afr J Lab Med. 2014;3(1), Art. #194, 8 pages. http://dx.doi.org/10.4102/ajlm.v3i1.194 10.4102/ajlm.v3i2.194 PMC4703335 26752335

[6] Mothabeng D, Maruta T, Lebina M, et al Strengthening laboratory management towards accreditation: the Lesotho experience. Afr J Lab Med. 2012;1(1), Art. #9, 7 pages. http://dx.doi.org/10.4102/ajlm.v1i1.9 10.4102/ajlm.v1i1.9 PMC5644518 29062729

[7] Yao K, McKinney B, Murphy A, et al Improving quality management systems of laboratories in developing countries: An innovative training approach to accelerate laboratory accreditation. Am J Clin Pathol. 2010;134(3):401–409. http://dx.doi.org/10.1309/AJCPNBBL53FWUIQJ 20716796 10.1309/AJCPNBBL53FWUIQJ

[8] Maruta T, Motebang D, Wanyoike J, et al Impact of mentorship on WHO-AFRO Strengthening Laboratory Quality Improvement Process Towards Accreditation (SLIPTA). Afr J Lab Med. 2012:1(1), Art. #6, 8 pages. http://dx.doi.org/10.4102/ajlm.v1i1.6 10.4102/ajlm.v1i1.6 PMC5644515 29062726

[9] Maruta T, Rotz P, Peter T Setting up a structured laboratory mentoring programme. Afr J Lab Med. 2013;2(1), Art. #77, 7 pages. http://dx.doi.org/10.4102/ajlm.v2i1.77 10.4102/ajlm.v2i1.77 PMC5637775 29043168

[10] Abimiku AG Building laboratory infrastructure to support scale-up of HIV/AIDS treatment, care, and prevention: In-country experience. Am J Clin Path. 2009;131(6):875–886. http://dx.doi.org/10.1309/AJCPELMG6GX6RQSM 19461097 10.1309/AJCPELMG6GX6RQSM

